# Prevalence and seasonal transmission of *Schistosoma haematobium* infection among school-aged children in Kaedi town, southern Mauritania

**DOI:** 10.1186/s13071-017-2284-4

**Published:** 2017-07-26

**Authors:** N’Guessan G C Gbalégba, Kigbafori D Silué, Ousmane Ba, Hampâté Ba, Nathan T Y Tian-Bi, Grégoire Y Yapi, Aboudramane Kaba, Brama Koné, Jürg Utzinger, Benjamin G Koudou

**Affiliations:** 1 Unité de Formation et de Recherche Sciences de la Nature, Université Nangui Abrogoua, 02 B.P. 801, Abidjan 02, Côte d’Ivoire; 2 Centre Suisse de Recherches Scientifiques en Côte d’Ivoire, 01 B.P. 1303, Abidjan 01, Côte d’Ivoire; 3 Unité de Formation et de Recherche Biosciences, Université Félix Houphouët-Boigny, 22 B.P. 582, Abidjan 22, Côte d’Ivoire; 4 Laboratoire de Parasitologie – Mycologie, Institut National de Recherches en Santé Publique, B.P, 695 Nouakchott, Mauritania; 5 Centre d’Entomologie Médicale et Vétérinaire (CEMV), Université Alassane Ouattara, Bouaké, Côte d’Ivoire; 6 Université Péléforo Gon Coulibaly, B.P, 1328, Korhogo, Côte d’Ivoire; 7 Swiss Tropical and Public Health Institute, P.O. Box, CH-4002 Basel, Switzerland; 8 University of Basel, P.O. Box, CH-4003 Basel, Switzerland; 9 Centre for Neglected Tropical Diseases, Liverpool School of Tropical Medicine, Pembroke Place, Liverpool, L3 5QA UK

**Keywords:** Prevalence, *Schistosoma haematobium*, Seasonal transmission, Snails, Water contact, Urban area, Mauritania

## Abstract

**Background:**

Mauritania is at the fringe of transmission of human schistosomiasis, which mainly occurs in the southern and southeastern parts of the country. This study aimed to assess the influence of rainfall seasonality on the prevalence of *Schistosoma haematobium* infection among school-aged children in Kaedi, southern Mauritania.

**Methods:**

Cross-sectional surveys (i.e. parasitological, malacological and observations on water-related human activities) were carried out in Kaedi between September 2014 and May 2015, during both the wet and dry seasons. A total of 2162 children aged 5–15 years provided a single urine sample that was subjected to *S. haematobium* diagnosis. Snails were sampled and checked for cercarial shedding. Water contact patterns of the local population were recorded by direct observation.

**Results:**

The prevalence of *S. haematobium* was 4.0% (86/2162, 95% confidence interval (CI): 3.2–4.9%) with a geometric mean egg count per 10 ml of urine of 3.7 (95% CI: 2.8–4.3). Being male (adjusted odds ratio (aOR) 1.78, 95% CI: 1.13–2.80), being at primary school (aOR 1.73, 95% CI: 1.04–2.87) and dry season (aOR 0.56, 95% CI: 0.35–0.89) were significantly associated with *S. haematobium*. Among 284 potential intermediate host snail specimens collected over the rainy and dry seasons, three species were identified: *Bulinus senegalensis* (*n* = 13) and *B. forskalii* (*n* = 161) in the rainy season, and *B. truncatus* (*n* = 157) in the wet season. No snail was shedding cercariae. On average, seven human water contacts were recorded per hour per observer over a 28-day observation period. Twelve types of water contact activities were identified among which, swimming/bathing was predominant (*n* = 3788, 36.9%), followed by washing clothes (*n* = 2016, 19.7%) and washing dishes (*n* = 1322, 12.9%). Females (*n* = 5270, 51.4%) were slightly more in contact with water than males (*n* = 4983, 48.6%). The average time spent in the water per person per day was 14.2 min (95% CI: 13.8–14.6 min). The frequency and duration of water contact followed a seasonal pattern.

**Conclusion:**

Our findings demonstrate a low prevalence and intensity of *S. haematobium* among school-aged children in Kaedi. Appropriate integrated control measures, including health education among at-risk communities and snail control may help to interrupt transmission of *S. haematobium* in Kaedi.

## Background

Schistosomiasis is one of the most widespread human parasitic diseases in terms of socioeconomic and public health impact in tropical and subtropical areas [1]. More than 250 million people are infected and the number of people needing preventive treatment in 2015 was estimated at 201 million [2, 3]. Approximately 85% of infections occur in sub-Saharan Africa and at least 90% of people requiring treatment for schistosomiasis live in Africa [4].

Various factors are responsible for the persistent transmission of schistosomiasis in sub-Saharan Africa. These include living in close proximity to water bodies, such as irrigation schemes and dams, as well as socioeconomic factors such as occupational activities, poverty and climate change [5–8]. The Sahel region is characterized by long periods of drought, which makes it difficult for its inhabitants to reach agricultural self-sufficiency. However, in the 1970s and 1980s, various irrigation systems have been installed throughout the region to enhance agriculture and food production [9]. Thus, important development programmes have been implemented, including the construction of large dams on the Senegal River [10, 11] that strongly influences cities located on the right borders of the river.

The current schistosomiasis control strategy is mainly based on preventive chemotherapy that is the periodic administration of the antischistosomal drug praziquantel to school-aged children and other high risk groups [12, 13]. Praziquantel reduces morbidity and might impact on transmission, but rarely eliminates infection [14, 15]. A major shortcoming to schistosomiasis control is the low coverage of preventive chemotherapy. In 2015, for example, only 26.8% of the people requiring preventive chemotherapy were administered praziquantel [3].

In Mauritania, transmission of schistosomiasis primarily occurs in the south and southeast where the prevalence might range from 1.3% to 90% [16, 17]. Shortly, after the construction of the Diama and Manantali dams, there was an outbreak of *Schistosoma mansoni* [18, 19]. Despite a mass distribution campaign of praziquantel in the country, transmission of schistosomiasis persists within the communities [17]. In Kaedi, most water contact activities of the local population occur in the Senegal River.

The current study was designed to deepen our understanding of the transmission of *S. haematobium* in Kaedi. Our findings are discussed in the frame of integrated schistosomiasis control and, indeed, current efforts that aim at elimination of schistosomiasis [20].

## Methods

### Study area and population

**Fig. 1:**
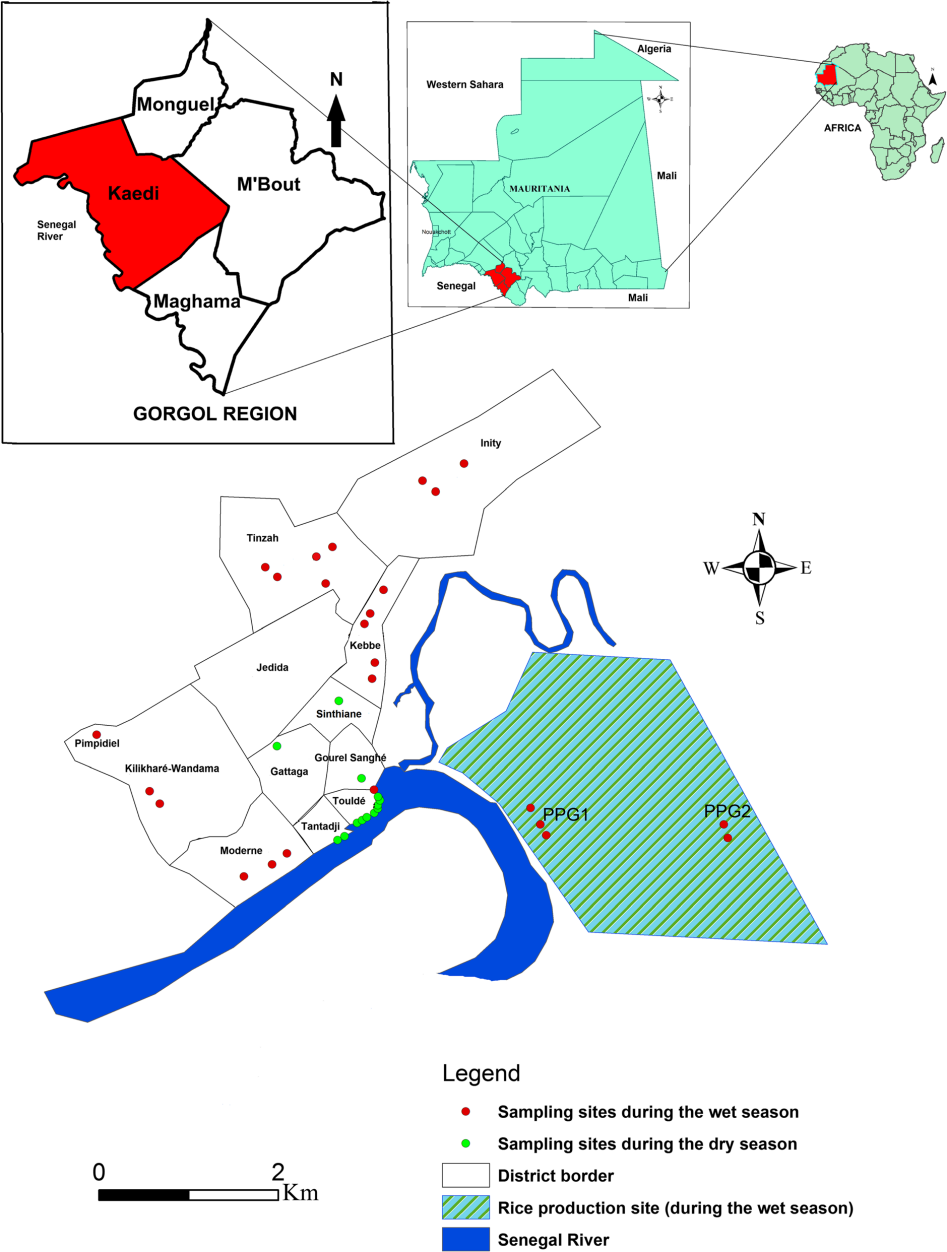
Map of the Islamic Republic of Mauritania, showing the study site and the distribution of snail sampling sites during the wet and dry season

The study was carried out in Kaedi town, southern Mauritania. Kaedi is located approximately 430 km from the capital Nouakchott. Kaedi is the main town of the Gorgol region (Fig. 1). The climate is Sahelian, with a 4-month-long rainy season (June-September) and a dry season for the rest of the year. The rainy season is characterized by short, at times violent rainfalls with a peak in August. During the dry season, there is a lack of precipitation with a somewhat cooler period from October to February and a hotter period from February to June. Minimum temperatures range from 12 °C in December to 30 °C in January, while maximum temperatures vary between 30 °C in January and 45 °C in June. The annual precipitation ranges from 300 to 500 mm. Kaedi is located along the Senegal River, which represents the main water body. The main occupation of the inhabitants is subsistence farming of vegetables, millet and sorghum. Livestock farming is done by mobile pastoralists. The wet season is the period of intensive agricultural activities, specifically rice paddy cultivation in "Périmètre Pilote du Gorgol 1" (PPG1) and "Périmètre Pilote du Gorgol 2" (PPG2), which are the two main rice growing areas (Fig. 1). All children between the ages of 5 and 15 years from randomly selected households were eligible for participation in the study, and hence, were invited to provide a single urine sample for the diagnosis of *S. haematobium*.

### Study design, sample size determination and household selection

**Fig. 2:**
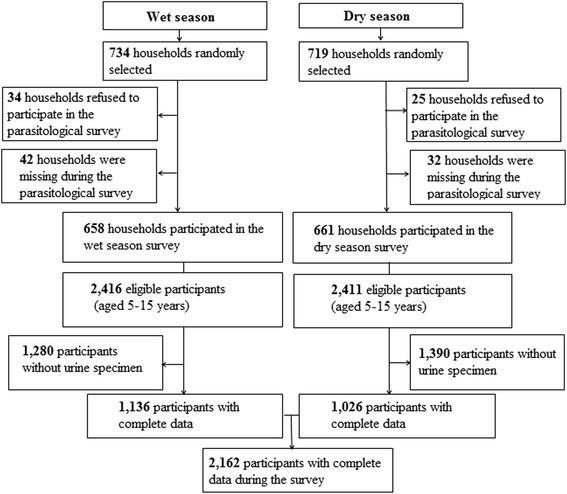
Flow chart showing the study cohort and compliance on the study participants in Kaedi, southern Mauritania, 2014/2015

Cross-sectional surveys (parasitological, malacological and observations of human water-related activities) were carried out simultaneously in September 2014, and in May 2015 during the wet season and dry season, respectively. The parasitological surveys were carried out in each season, using a community-based approach. Households were selected randomly from 11 districts of the city. The number of households per district was proportionally allocated according to the population size of each district (Fig. 2). The sample size (*n*) was adjusted to 728 households obtained by using the formula *n* = $\frac{\updelta^2\times P\left(1-P\right)\times C}{i^2}$, where *δ* is the standard deviation (1.96), *P* is the expected prevalence (35%) based on Ouldabdallahi et al. [16], *i* is the precision or margin of the error (5%) and *C* is the correction coefficient (*C* = 2).

### Urine collection and microscopic examination

Participants were invited to provide a single urine sample in a 15 ml empty sterile container. Urine samples were collected between 10:00 and 14:00 h directly at the households. Urine sample were subjected to the filtration method, as described elsewhere [21]. In brief, 10 ml of vigorously shaken urine were gently pressed through a filter (12-μm polycarbonate filter). The filter was placed on a microscope slide and a drop of Lugol’s iodine solution was added before quantitative examination under the microscope by two experienced laboratory technicians at an X 100 magnification. Eggs were counted and recorded as number of eggs/10 ml of urine. Infection intensity was classified as light (< 50 eggs/10 ml of urine) or heavy (≥ 50 eggs/10 ml of urine), according to guidelines put forth by the World Health Organization (WHO) [22]. For quality control, 10% of the slides were re-examined by a senior laboratory technician.

### Snail sampling

A preliminary investigation in Kaedi was conducted to identify sites where people were involved in water contact activities. Owing to the Sahelian climate, ponds are scarce in Kaedi. This allowed us to identify and visit all available water-contact points (*n* = 39). Snails were collected during five consecutive days both in the dry and wet season at each water-contact site (Fig. 1) by two previously trained field enumerators using standard snail scoops or, occasionally, by hand collection. Sampling time was fixed at 15 min per collection site [23]. The geographical coordinates of the visited sites were determined using a global positioning system (GPS MAP 62, Garmin; Olathe, KS, USA). The snail density was expressed as the number of snails collected per collector during 15 min at each site [24]. Snails were transfered in small boxes designed for their transportation from the field to the laboratory. In the Kaedi Health Centre laboratory, snails were rinsed with tap water and identified to genus or species level, according to readily available identification keys [25, 26] and examined for schistosome infection by cercarial shedding. Each snail specimen identified as potential intermediate host of schistosomiasis was placed in a small plastic cup containing 20 ml of distilled water and exposed to direct artificial light (36 W neon) for 20–35 min. Patent infections in snails were subsequently investigated using a binocular lens against a dark background. The ratio of the number of snails shedding *Schistosoma* spp. cercariae to the total number of snails tested represented the prevalence. During each season, all intermediate host snails were tested up to a full cycle of 30 days.

### Human water-contact activities

Direct observations were made with an emphasis on the behaviour of community members in order to understand how they might become infected with schistosomes. These observations took place in close proximity to the river widely used by people in the city. Two sites were selected each season. Site 1 was a rice perimeter at PPG1, while site 2 was around Senegal River. During the dry season, PPG1 site was replaced by another site on the river bank (because there was no rice cultivation during this period), while site 2 was maintained. Behaviours of community members during water contact were recorded each day from 06:00 to 19:00 h during each season by trained observers [27]. A supervisor randomly visited the observers several times per day to make sure that they adhered to standard protocols [28]. Each individual who was in contact with surface water was identified by the observer who then recorded the sex, the type of water-contact activity, the part of the body in contact with the water and the start and end time the water was contacted. Additionally, the age was estimated by considering three age groups: (i) < 10 years; (ii) 10–15 years; and (iii) > 15 years.

### Statistical analysis

Data were entered with EpiData (EpiData Association; Odense, Denmark) and analyzed using Stata version 14.1 (Stata Corporation; College Station, TX, USA) [29]. Prevalence of infection was estimated as a proportion and compared *via* Pearson *χ* ^2^ test. Data summaries were made using descriptive statistics. The number of *S. haematobium* eggs counted were transformed to log_10_ (x + 1) values to normalize the distribution of the residuals values for statistical analyses. One-way ANOVA or Student’s *t*-test was used to check any statistical difference in mean egg counts for *S. haematobium* between seasons, sex and age-groups. Bivariate and multivariable logistic regression analyses were employed to determine the association between dependent and independent variables. Factors with *P-*value < 0.05 as identified at bivariate level were considered for the final model. An odds ratio (OR) with 95% confidence interval (CI) was used to measure the strength of association between *S. haematobium* infection status and co-variables. A 5% level of significance was applied to all statistical tests.

## Results

### Characteristics of the study participants

The demographic characteristics of study participants during both wet and dry season in Kaedi town are summarized in Table 1. For the parasitological survey, a total of 4827 individuals aged 5–15 years were recruited. However, only 44.7% (*n* = 2162) of the children provided urine samples. The sex ratio (male:female) was 0.97 with 1064 (49.2%) being male. The mean age of the study participants was 9.3 years. The majority of recruited children who had complete data records (1335/2162; 61.7%) attended primary school.

**Table 1:** Characteristics of study participants in Kaedi, southern Mauritania, 2014/2015

Variable	Frequency	%	*P*-value
Sex			
Male	1064	49.2	0.373
Female	1098	50.8	
Sex ratio (males:females)	0.97		
Age group (in years)			
5–7	761	35.2	< 0.001
8–11	783	36.2	
12–15	618	28.6	
Mean age	9.3		
Education level			
No education	446	20.6	< 0.001
Koranic school	256	11.8	
Primary school	1335	61.7	
Secondary school	125	5.8	
Relationship to the household head			
Child	1468	67.9	0.282
Other relatives	663	30.7	
Without relatives	31	1.4	
Household head occupation			
Officials^a^	209	14.0	0.502
Non-officials^b^	943	63.3	
Unemployed	339	22.7	
Household head education level			
No education	449	30.1	0.470
Koranic school	515	34.5	
Primary school	252	16.9	
Secondary school	209	14.0	
Higher academic level	67	4.5	
Habitation zone^c^			
Zone 1	775	35.8	0.634
Zone 2	767	35.5	
Zone 3	620	28.7	
Season			
Wet	1026	47.5	0.002
Dry	1136	52.5	
Total	2162	100.0	

^a^Including teachers, medical staff and administration staff

^b^Including famers, traders and craftsmen

^c^Habitation in the study site was divided in zones according to the distance to the Senegal River: zone 1, < 200 m; zone 2, 201–500 m; zone 3, > 500 m

### Prevalence, geometric means of egg counts and factors associated with *S. haematobium*

**Fig. 3:**
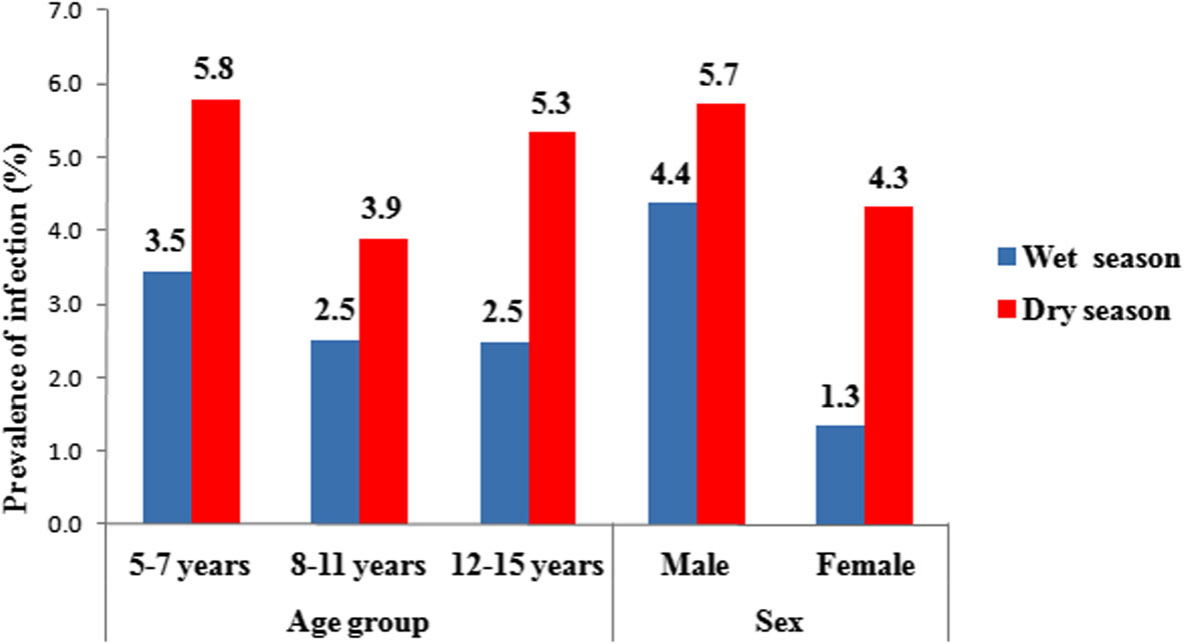
Prevalence rate of *S. haematobium* infection, stratified by sex, age group and season in Kaedi, southern Mauritania, 2014/ 2015

The prevalence and the geometric mean of egg counts (GMEC) of *S. haematobium* according to season and demographic characteristics are presented in Table 2. Of the 2162 urine samples examined, 86 were positive (4.0%, 95% CI: 3.2–4.9%) for *S. haematobium* eggs. The prevalence was significantly higher in males (54/1064; 5.1%) than females (32/1098; 2.9%) (*χ* ^2^ = 6.60, *P* = 0.010) (Fig. 3). There was no statistically significant difference between infection rates recorded within different age groups (*χ* ^2^ = 2.38, *P* = 0.304), according to the education attainment of the parents/guardians (*χ* ^2^ = 7.50, *P* = 0.111), the relationship with the head of households (*χ* ^2^ = 1.30, *P* = 0.520), occupation of household heads (*χ* ^2^ = 5.15, *P* = 0.076) and habitation zone (*χ* ^2^ = 3.82, *P* = 0.148). The prevalence of *S. haematobium* infection was significantly higher during the dry season (57/1136, 5.0%) compared to the rainy season (29/1026, 2.8%), (*χ* ^2^ = 6.76, *P* = 0.009). The overall GMEC was 3.7 eggs/10 ml of urine. In relation to sex, males (3.5 eggs/10 ml of urine) and females (3.4 eggs/10 ml of urine), had similar GMEC (*t* = 1.60, *P* = 0.934). The intensity of infection varied significantly according to education level (*F*_(3,82)_ = 3.78, *P* = 0.013) and habitation zone (*F*_(2,83)_ = 3.15, *P* = 0.048) but did not follow a seasonal variation (*t* = 1.76, *P* = 0.081). Indeed, children from Koranic schools had the highest GMEC (8.9 eggs/10 ml of urine, 95% CI: 3.6–30.9), while the lowest GMEC was recorded among children from secondary school (2.1 eggs/10 ml of urine, 95% CI: 1.0–2.9). The GMEC decreased according to age with 3.8 (95% CI: 2.6–5.9), 3.7 (95% CI: 2.7–5.6) and 2.8 (95% CI: 2.0–4.2) eggs/10 ml of urine in 5–7, 8–11 and 12–15 years age groups, respectively.

**Table 2:** Prevalence, geometric mean of eggs counts and factors associated with frequency of *Schistosoma haematobium* in Kaedi, southern Mauritania, 2014/2015

Variable	Frequency of *S. haematobium*			Bivariate	Multivariate	GMEC (95% CI)
	*N*	Positive	Prevalence (95% CI)	cOR (95% CI)	aOR (95% CI)	
Overall	2162	86	4.0 (3.2**–**4.9)			3.67 (2.80**–**4.31)
Sex						
Female	1064	54	5.1 (3.9**–**6.6)	1.00	1.00	3.50 (2.64**–**4.85)
Male	1098	32	2.9 (2.1**–**4.1)	1.78 (1.14**–**2.78)	1.75 (1.11**–**2.77)	3.38 (2.58**–**4.76)
*P*-value			0.010			0.114^b^
Age group (in years)						
5**–**7	761	35	4.7 (3.4**–**6.5)	1.00		3.82 (2.64**–**5.87)
8**–**11	783	26	3.2 (2.2**–**4.7)	1.40 (0.84**–**2.35)		3.74 (2.70**–**5.58)
12**–**15	618	25	4.0 (2.7**–**5.9)	1.14 (0.68**–**1.93)		2.80 (2.00**–**4.18)
*P*-value			0.436			0.522^a^
Education level						
No education	446	25	5.6 (3.8**–**8.2)	1.00	1.00	4.17 (2.74**–**6.95)
Koranic school	256	11	4.3 (2.4**–**7.6)	1.32 (0.64**–**2.73)	1.82 (0.86**–**3.85)	8.93 (3.56**–**30.87)
Primary school	1335	45	3.4 (2.5**–**4.5)	1.70 (1.03**–**2.81)	1.79 (1.07**–**2.99)	2.91 (2.34**–**3.70)
Secondary school	125	5	4.0 (1.7**–**9.3)	1.42 (0.53**–**3.80)	1.56 (0.57**–**4.25)	2.13 (1.00**–**2.96)
*P*-value			0.217			0.013^a^
Relationship with household head						
Child	1468	59	4.0 (3.1**–**5.2)	1.00		3.32 (2.63**–**4.26)
Other relatives	663	27	4.1 (2.8**–**5.9)	0.98 (0.62**–**1.57)		4.17 (2.69**–**7.17)
Without relatives	31	0	–	**-**		–
*P*-value			0.520			0.329^b^
Household head’s education level						
No education	449	31	6.9 (4.7**–**9.7)	1.00		4.17 (3.09**–**6.17)
Koranic school	515	23	4.5 (2.9**–**6.6)	0.58 (0.91**–**2.76)		5.10 (3.89**–**7.38)
Primary school	252	21	8.3 (5.2**–**12.4)	0.81 (0.46**–**1.45)		3.70 (2.91**–**5.26)
Secondary school	209	9	4.3 (2.0**–**8.0)	1.65 (0.77**–**3.53)		3.03 (2.00**–**6.95)
Higher academic level	67	2	3.0 (0.4**–**10.4)	2.41 (0.56**–**10.31)		7.38 (3.78**–**19.49)
*P*-value			0.111			0.334^a^
Household head’s occupation						
Non-officials^c^	943	61	6.5 (5.0**–**8.2)	0.48 (0.25**–**0 .93)	0.49 (0.25**–**0.95)	3.39 (2.70**–**5.41)
Officials^d^	209	14	6.7 (3.7**–**11.0)	0.46 (0.21**–**1.05)	0.46 (0.20**–**1.03)	2.97 (2.70**–**7.38)
Unemployed	339	11	3.2 (1.6**–**5.7)	1.00	1.00	3.38 (2.13**–**7.84)
*P*-value			0.076			0.476^a^
Habitation zone						
Zone 1	775	39	5.0 (3.7**–**6.8)	0.60 (0.34**–**1.04)		4.42 (3.32**–**6.75)
Zone 2	767	28	3.6 (2.5**–**5.2)	0.83 (0.46**–**1.51)		2.53 (1.87**–**3.63)
Zone 3	620	19	3.0 (2.0**–**4.8)	1.00		3.70 (2.48**–**6.05)
*P*-value			0.148			0.048^a^
Season						
Wet	1026	29	2.8 (2.0**–**4.0)	1.00	1.00	4.80 (3.01**–**8.48)
Dry	1136	57	5.0 (3.9**–**6.5)	0.55 (0.35**–**0.87)	1.71 (1.07**–**2.74)	5.10 (3.25**–**8.84)
*P*-value			0.009			0.081^b^

Factors with *P-*value <0.05 were identified at bivariate level and were considered for the multivariate model

^a^Obtained by ANOVA

^b^Obtained by Student’s *t*-test

^c^Famers, traders, craftsmen

^d^Teachers, medical staff, administrators

*Abbreviations*: *aOR* adjusted odds ratio, *CI* confidence interval, *cOR* crude odds ratio, *GEMC* geometric mean of egg count, *N* number examined

The results of bivariate and multivariable analysis for the factors associated with *S. haematobium* infection are presented in Table 2. In the bivariate analysis, male (crude OR, (cOR) 1.78, 95% CI: 1.14–2.78), attending primary school (cOR 1.70, 95% CI: 1.03–2.81), no officials (e.g. farmers, traders and craftsmen) as household head’s occupation (cOR 0.48, 95% CI: 0.25–0.93) and dry season (cOR 0.55, 95% CI: 0.35–0.87) were significantly associated with *S. haematobium*. However, in the multivariable logistic regression analysis, only sex, educational attainment and season were significantly associated with *S. haematobium*. Males were nearly two times more likely to be infected with *S. haematobium* than females (adjusted OR, (aOR) 1.75, 95% CI: 1.11–2.77). Children at primary school were nearly two times more likely to be infected than those who did not go to school (aOR 1.79, 95% CI: 1.07–2.99). The dry season (aOR 1.71, 95% CI: 1.07–2.74) appeared to be a high risk period of *S. haematobium* infection compared to the rainy season.

### Seasonal variation of snail abundance and infectivity

A total of 39 sites in four habitats (i.e. rice fields, river bank, drain and temporary ponds) were surveyed, during the wet season (*n* = 26, 66.6%) and during the dry season (*n* = 13, 33.4%). During the dry season, 11 of the 13 sites were located on the Senegal River bank, whilst, in the wet season, only two of the 26 sites were located at this same place. Overall, 331 snails were collected during both seasons, 174 (52.6%) and 157 (47.4%) during the wet and dry seasons, respectively. Based on shell morphology, using readily available field identification keys, 13 (3.9%), 157 (47.4%) and 161 (48.6%) of the snails were identified as *Bulinus senegalensis*, *B. truncatus* and *B. forskalii*, respectively. These three species have been identified as intermediate host snails of schistosomiasis. The snail abundance varied according to each species from dry to rainy season (*P* < 0.05). At the ecological level, snails were found in all habitats except in rice fields. *Bulinus senegalensis* (collected in drains) and *B. forskalii* (collected in temporary ponds) were collected only during the wet season, whilst *B. truncatus* were collected (only on the river bank) exclusively during the dry season. On the river bank, snails were abundant in areas under less human activities and with little vegetation. Most of the collected snails (*n* = 298, 90.0%) were tied to submerged plastic bags. Out of 284 snails subjected to shedding, no *S. haematobium* cercariae were found (Table 3).

**Table 3:** Seasonal inventory of the malacological fauna in Kaedi, southern Mauritania, 2014/2015

Biotope	Species	Wet season			Dry season		
		*n* (%)	*n* tested	IR	*n* (%)	*n* tested	IR
Irrigation canal	*B. senegalensis*	13 (7.5)	13	0	0 (0)	0	**–**
River bank	*B. truncatus*	0 (0)	0	**–**	157 (100)	154	0
Temporary pond	*B. forskalii*	161 (92.5)	117	0	0 (0)	0	**–**
Total		174 (100)	130	0	157(100)	154	0

*Abbreviations*: *IR* infection rate; n, number of snails collected; n tested, number of snails tested

### Water-related activities and duration

**Fig. 4:**
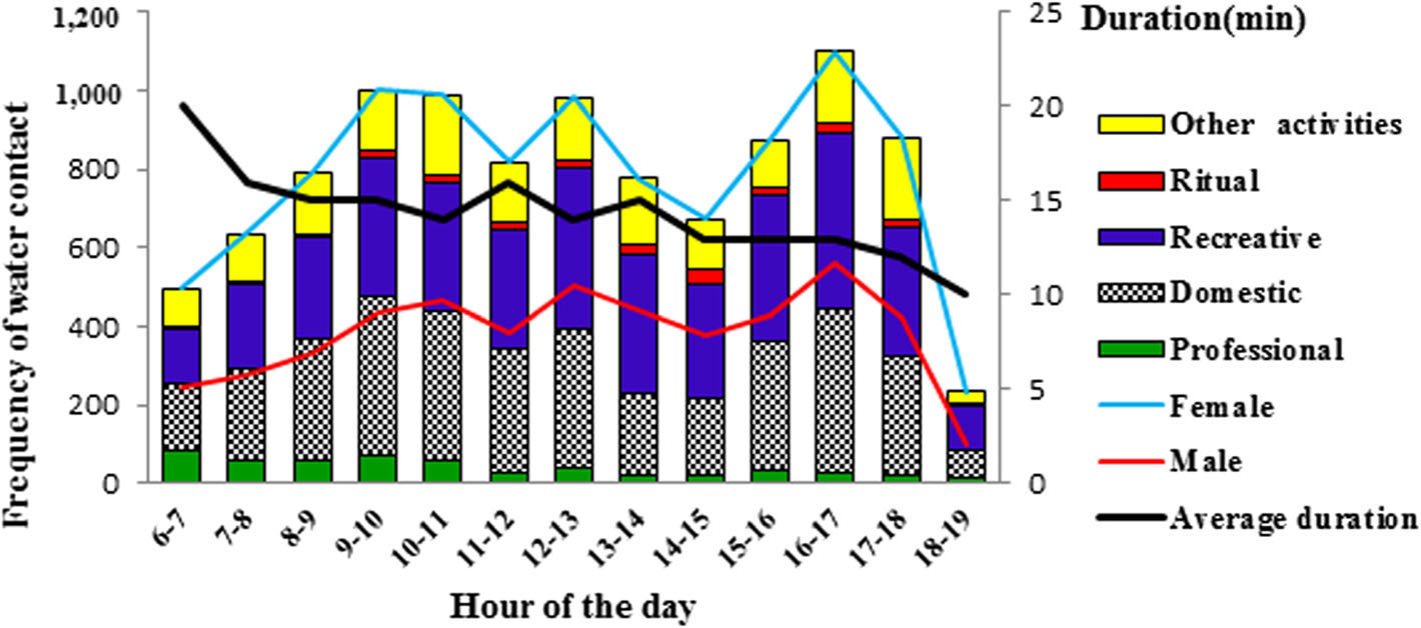
Frequency of water contact by hour, category of activity, sex and mean duration of contact in Kaedi, southern Mauritania, 2014/2015

A total of 10,253 human-water contacts during 28 observation days, corresponding to 145,710 min, were recorded (Table 4). The frequency of water-contact activities was higher during the wet season (*n* = 5707, 55.6%) compared to the dry season (*n* = 4546, 44.4%) (*P* < 0.001). Twelve water-contact activities were recorded during the study. These activities were categorized in five types: (i) professional (e.g. fishing), (ii) domestic, (iii) recreational, (iv) ritual and (v) other diverse activities. Regarding categories of activities, recreational activities (*n* = 3909, 38.1%) were the main activities, followed by domestic activities (*n* = 3697, 37.4%), other activities include animal washing/watering, washing motorized machines (*n* = 1888, 18.4%), professional (*n* = 533, 5.2%) and ritual (*n* = 226, 2.2%) activities. During the dry season, rice cultivation frequency was zero, because the paddy cultivation took place only during the wet season in PPG1 and PPG2 sites. The majority of professional activities (*n* = 453, 85%) took place during the wet season. Concerning the recreational activities, the frequency recorded was three times higher during the wet season (*n* = 3091, 81.6%) compared to the dry season (*n* = 697, 18.40%), (*χ* ^2^ = 6.11, *P* = 0.013). Regarding sex, females (*n* = 5270, 51.4%) were more in contact with water than their male counterparts (*n* = 4983, 48.6%) (*P* < 0.001) and spent more time (average duration 15.9 min (95% CI: 15.2–16.7 min) in water than males (average duration 14.2 min (95% CI: 13.1–15.3 min) **(**Fig. 4). Female contacts with water were mainly related to domestic activities (55.7% of contacts), followed by recreational activities (37.8% of the contacts). In contrast, males were firstly attracted by water for recreational activities (38.8% of contacts), such as swimming, followed by other various activities such as washing/watering livestock and washing motorized machines (31.6% of contacts). However, according to age, adolescents and adults (> 15 years, *n* = 4385, 42.8%) were most in contact with water, followed by those aged 10–15 years (*n* = 3653, 35.6%) and those < 10 years old (*n* = 2215, 21.6%). The average time spent in the water per person per day was 14.2 min (95% CI: 13.8–14.6 min). This mean was higher during the wet season (18.8 min; 95% CI: 18.2–19.4 min) compared to the dry season (8.5 min, 95% CI: 8.3–8.7 min). The intensity of contacts with water varied according to sex and the period of the day. Most intense water contacts occurred for males and females during three distinct peaks; first in the morning between 9:00 and 10:00 h, then around noon (12:00–14:00 h) and in the late afternoon (16:00–17:00 h) (Fig. 4).

**Table 4:** Overview of frequency, duration and average duration of water contact in Kaedi, southern Mauritania, 2014/2015

Activities	Wet season			Dry season		
	Frequency	Total duration (min)	Average duration (min) (95% CI)	Frequency	Total duration (min)	Average duration (min) (95% CI)
Professional						
Fishing	178	13,519	75.9 (67.4**–**84.5)	156	2080	13.4 (11.5**–**15.1)
Rice farming	301	3423	11.4 (10.5**–**12.2)	**–**	**–**	**–**
Gardening	41	1045	25.5 (19.0**–**31.9)	52	286	5.5 (4.9**–**6.1)
Domestic						
Fetching water	223	971	4.4 (3.7**–**5.0)	136	665	4.9 (4.2**–**5.5)
Washing clothes	581	21,792	37.5 (34.9**–**40.1)	1435	14,016	9.8 (9.5**–**10.0)
Washing dishes	660	12,221	18.5 (17.4**–**19.6)	662	7112	10.7 (10.2**–**11.3)
Recreative						
Swimming (bathing)	3091	47,336	15.3 (14.8**–**15.8)	697	5852	8.4 (8.0**–**8.8)
Ritual						
Ablution / worship of water	216	1026	4.8 (0.8**–**8.7)	10	56	5.6 (4.0**–**7.2)
Micturate / drinking	27	38	1.4 (1.1**–**1.7)	160	316	2.0 (1.6**–**2.3)
Other activities						
Animals washing or watering	223	4130	18.6 (14.1**–**23.1)	314	2542	8.1 (7.5**–**8.7)
Walking	108	336	3.1 (2.4**–**3.8)	238	538	2.3 (2.0**–**2.5)
Washing motorized machine	58	1254	21.6 (17.0**–**26.2)	182	2499	13.7 (12.7**–**14.8)
(Dis) embarking	**–**	**–**	**–**	504	2658	5.3 (5.1**–**5.4)
Total	5707	107,091	18.8 (18.2–19.4)	4546	38,619	

*Abbreviation*: *CI* confidence interval

## Discussion

The present study extends our understanding of the seasonal transmission of *S. haematobium* in the urban area of Kaedi, southern Mauritania. As the Senegal River is the only source of permanent water supply for the Kaedi community, it is exceedingly difficult to prevent the community from contacting this essential source of water on a daily basis for various uses (bathing, swimming, fishing and other domestic uses). This water constitutes the main transmission foci of schistosomiasis in Kaedi.

The prevalence of *S. haematobium* infection among school-aged children was found to be 4.0%, which classifies Kaedi town as a low endemic area [22]. This observed prevalence is low compared to previous studies with most studies conducted in different parts of southern Mauritania, known to be the main endemic area for schistosomiasis [16, 17, 30]. Our observed prevalence is considerably lower than that reported from other studies conducted in different parts of the Sahel, such as Burkina Faso (8.8%) [31], Mali (7.6%) [32] and Sudan (8.6%) [33]. Various ecological features might explain these differences, as they influence the transmission of schistosomiasis [34]. The low prevalence of *S. haematobium* in the present study might also be attributable to the preventive chemotherapy campaigns conducted recently in the southern part of the country, including the Gorgol region by the national schistosomiasis control programme [17]. In the current study, males were nearly two times more likely to be infected with *S. haematobium* than females, though females were more often in contact with water than males. This is explained by gender-specific water-contact activities. The risk of infection among girls was lower than boys. Indeed, during swimming (the predominant water contact by boys), boys exposed their whole bodies in the water, while girls usually only exposed their legs and hands into the water, mainly in relation to carrying out domestic activities (e.g. fetching water, washing clothes and dishes, etc.) Moreover, during the wet season, people, particularly boys, work in rice paddies. The higher prevalence in boys compared to girls confirms other reports for *S. haematobium* infection in Mauritania [16, 17], Senegal [35], Benin [36] and Ethiopia [37]. However, this result is in contradiction with similar studies carried out in Uganda [38] and Nigeria [39], where females showed higher infection rates than males.

The age-related prevalence showed that all age groups were at similar risk to be infected. However, children aged 5–7 years were slightly more affected, probably because they are frequently involved in water-contact activities (e.g. swimming, washing or watering animals) and were perhaps not yet targeted by preventive chemotherapy.

Children at primary school were nearly two times more likely to be infected than those from the same age who did not go to school. How can this unexpected finding be explained? In Kaedi, parents who wish their children to attend the French-language training programmes, send their children to schools in Senegalese villages at the other side of the Senegal River. Crossing the river takes about 2 min in a motorized canoe. Every school day, around 13:00 h, when children return from school in neighbouring Senegal and get out of the canoe, they take a recreational swim before going home. We hypothesis that this specific behaviour is the main risk factor for acquiring *S. haematobium* infection.

The average intensity of *S. haematobium* infection (3.7 eggs/10 ml) recorded was low. Indeed, it is less than that obtained in rural area of southern Mauritania [16]. Our finding showed that GMEC decreased with age. Similar observations have been reported by Dalton & Pole [40], who observed lower GMEC among elderly people in a village situated in close proximity to a man-made lake in Ghana. This could be due to the development of partial immunity as reported elsewhere [41, 42].

A deeper understanding of snail ecology and the ecological mechanisms affecting transmission can play an important role in integrated control of schistosomiasis and other snail**-**borne diseases [43]. Three species of snails that are known as intermediate hosts for *S. haematobium* (i.e. *B. truncatus*, *B. senegalensis* and *B. forskalii*) were collected from the study area. *Bulinus truncatus* and *B. forskalii* species were reported recently for the first time on the Mauritanian side of the Senegal River, while *B. forskalii* has not previously been identified as an intermediate host of *S. haematobium* in Mauritania [16]. The high occurrence of *B. truncatus* found during the dry season, coupled with the high prevalence of infection during this season suggests that active transmission of *S. haematobium* occurs in Kaedi during the dry season. During the dry season, water movements are expected to be low, thus offering a stable environment for snails to proliferate without being washed away [44]. *Bulinus truncatus* was reported in other studies carried out in the Senegal River basin in Mauritania [16]. *Bulinus senegalensis* were collected only during the wet season in an irrigated canal because in Kaedi, paddies rice cultivation is only pursued in the wet season. Previous studies reported that *B. senegalensis* could survive for 6–8 months when ponds are dry [45]. *Bulinus senegalensis* was reported for the first time on the Mauritanian side of the Senegal River during 2005–2006 and laboratory experiments indicated its role as an intermediate host for *S. haematobium* in Mauritania [16]. *Bulinus senegalensis* was already involved in *S. haematobium* transmission in the Senegal River basin [46, 47]. This species is endemic in West Africa, mainly in Sahelian area and was recorded in the Union for Conservation of Nature (IUCN) Red List of threatened species in 2010 [48]. At the ecological level, the present study revealed that, intermediate host snails of *S. haematobium* could breed in both natural (river bank) and man-made habitats (drains). However, out of the 284 snails tested, none was found to be shedding cercariae. The absence of infected snails could indicate that there is a low snail-*S. haematobium* compatibility, or the regular implementation of mass drug administration in the study area [17]. Based on the seasonal fluctuation of snails, focal mollusciciding at the beginning of the rainy season should be considered in an effort to eliminate the disease [20].

Water-contact studies are useful for determining major human activities that create a high risk of exposure to schistosome infection in areas where it occurs [49]. There is much scientific evidence that socio-demographic variables and contact with unsafe water are associated with schistosomiasis [50]. Our findings show that swimming/bathing was the main activity, followed by washing clothes and dishes. This result is in line with another study conducted in the Senegal River basin [28]. In sub-Saharan Africa, the most important water contact activity among children is swimming with a frequency depending on the variability of ambient temperature and humidity [50]. Laundry, bathing and recreational swimming are the activities that are causing the most exposure to cercaria-infested water, while the collection of water for drinking seems to pose a minor threat because it does not involve the immersion of large body parts, for long periods [51]. Changing the frequency and/or nature of water contact of communities living in Kaedi is not a feasible means of preventing schistosomiasis transmission. The same conclusion was drawn before for a Mauritanian village [52]. Limiting access to the river as a strategy to reduce transmission, during high-risk periods, as proposed in Sourou Valley of Burkina Faso [53] may not be feasible in Kaedi. Females had greater contact with water than males. This can be explained by the fact that females had simultaneously more than one water-contact activity compared to males. This result is in agreement with findings reported in villages of northern Senegal [54].

The average time spent in the water per person per day in the present study was 14.2 min. Other studies also pursuing direct observations reported considerably shorter water contact activities; 5.5 min in a Mauritanian village [52], 2.4 min in Kenya [55] and 4.3 min in Senegal [28]. Though it is known that the peak of cercarial shedding is around noon [50, 56], a long duration of contact with water is considered the more important risk factor for exposure to *S. haematobium* than frequency of the water contact [39]. The number and duration of daily contacts with water plays an important role in determining the relative risk of infection.

Our study has several limitations that should be taken into consideration when interpreting the results. First, the current level of infection may be underestimated. Indeed, during the door–to-door sample collection, some urine samples were provided earlier in the morning (due to logistical reasons) because several participants (schoolchildren) went to school. Normally, urine samples are collected between 10:00 and 14:00 h when schistosome eggs are most abundant in urine. In addition, direct observations tend to under-report water contacts taking place outside the observation periods or outside the selected sites [56].

## Conclusion

Urogenital schistosomiasis was found to be present at very low prevalence and intensity, with seasonal variation in Kaedi, southern Mauritania. Based on our findings, Kaedi is an area of low endemicity for *S. haematobium.* Preventive chemotherapy with praziquantel should be implemented in Kaedi during the dry season. In addition, management of the river bank by elimination of plastic bags, focal mollusciding, provision of safe water supply, sanitary facilities and implementation of continuous health education campaigns will be essential to eliminate *S. haematobium*. Agricultural activities performed during the wet season in the Gorgol throughout intensive rice paddy cultivation need to be also investigated to understand the contribution of anthropogenic factors in the transmission of *S. haematobium.*

## Abbreviations

aOR: Adjusted odds ratio
CI: Confidence interval
cOR: Crude odds ratio
GMEC: Geometric mean of egg count
GPS: Global positioning system
IDRC: International Development Research Centre
IUCN: International Union for Conservation of Nature
WHO: World Health Organization.
